# Management of children's urinary tract infections in Dutch family practice: a cohort study

**DOI:** 10.1186/1471-2296-8-9

**Published:** 2007-03-13

**Authors:** Mirjam Harmsen, Michel Wensing, Jozé CC Braspenning, René J Wolters, Johannes C van der Wouden, Richard PTM Grol

**Affiliations:** 1 Centre for Quality of Care Research (WOK), Radboud University Nijmegen Medical Centre, PO Box 9101 (114 kwazo), 6500 HB Nijmegen, The Netherlands; 2 Department of General Practice, Erasmus MC – University Medical Center Rotterdam, PO Box 1738, 3000 DR, Rotterdam, The Netherlands

## Abstract

**Background:**

Optimal clinical management of childhood urinary tract infections (UTI) potentiates long-term positive health effects. Insight into the quality of care in Dutch family practices for UTIs was limited, particularly regarding observation periods of more than a year. Our aim was to describe the clinical management of young children's UTIs in Dutch primary care and to compare this to the national guideline recommendations.

**Methods:**

In this cohort study, all 0 to 6-year-old children with a diagnosed UTI in 2001 were identified within the Netherlands Information Network of General Practitioners (LINH), which comprises 120 practices. From the Dutch guideline on urinary tract infections, seven indicators were derived, on prescription, follow-up, and referral.

**Results:**

Of the 284 children with UTI who could be followed for three years, 183 (64%) were registered to have had one cystitis episode, 52 (18%) had two episodes, and 43 (15%) had three or more episodes. Another six children were registered to have had one or two episodes of acute pyelonephritis. Overall, antibiotics were prescribed for 66% of the children having had ≤ 3 cystitis episodes, two-thirds of whom received the antibiotics of first choice. About 30% of all episodes were followed up in general practice. Thirty-eight children were referred (14%), mostly to a paediatrician (76%). Less than one-third of the children who should have been referred was actually referred.

**Conclusion:**

Treatment of childhood UTIs in Dutch family practice should be improved with respect to prescription, follow-up, and referral. Quality improvement should address the low incidence of urinary tract infections in children in family practice.

## Background

Awareness of the importance of timely diagnosis and treatment of childhood urinary tract infections (UTIs) is growing. Without timely treatment, renal scarring can occur [1], which is likely to affect approximately 5–15% of young children with a UTI [2-5]. Renal scarring is associated with serious health problems in later life, such as hypertension, complications during pregnancy, and renal failure [1]. Optimal clinical management of childhood UTI potentiates long-term positive health effects. Therefore, guidelines across different countries advocate an active approach concerning prescription, follow-up, and referral [6-9].

The guideline on UTIs of the Dutch College of General Practitioners (DCGP) states that every childhood UTI should be treated with antibiotics because of the risk of renal scarring. Amoxicillin/clavulanic acid or co-trimoxazole are the medications of choice in an attempt to reach effective tissue levels and to maximize the chance of the most effective medicine until test results are available. The follow-up recommendation takes into account that young children may not express their complaints clearly, while they have a high risk of renal scarring. The referral recommendations are based on the patients' age and sex, which predict the probability of anatomical abnormalities of the urinary tract system [9].

The one-year incidence of UTIs in 0 to 6-year-old children in Dutch family practices averages for girls 25.7 and for boys 4.5 per 1000 life-years [10]. Insight into the quality of care for UTI was limited, particularly regarding observation periods of more than a year. We have acquired the necessary prospective data to provide insight into the primary-care-based management of childhood UTIs in the Netherlands. We aimed to describe the clinical management of young children's UTIs in Dutch primary care and compare this to the national guideline recommendations.

## Methods

### Design and setting

A prospective cohort study in Dutch family practice was performed.

### Study population

We identified all children 0–6 years old (born between 1994 and 2001) with UTI diagnosed in 2001 within the Netherlands Information Network of General Practice (LINH). The LINH network contains 120 practices and is representative of the Dutch population of patients, family practitioners, and types of practices [11]. Informed consent was arranged within the network (general board of the National Institute for Health Services Research (NIVEL), general board of the Centre for Quality of Care Research (WOK), general board of the Dutch College of General Practitioners (NHG), and the general board of the National Association of General Practitioners (LHV)). According tothe Dutch Central Committee on Research Involving Human Subjects (CCMO) regulations only research in which the study participant has to be physically present during the study is subject to the Medical Research Involving Human Subjects Act (WMO) and therefore ethical approval is not required for studies that use patient databases.

The International Classification of Primary Care (ICPC) [12] defines UTI as acute pyelonephritis (ICPC code U70) or cystitis (ICPC code U71). These definitions imply that UTI was diagnosed by urine testing, not just suspected or assumed.

### Measurements

Although there is no specific DCGP guideline on UTIs in children, the Dutch UTI guideline does include specific recommendations for children [13]. We derived seven clinical indicators of appropriate performance. Two indicators focused on medication, one on follow-up, and four on referring (Table 1).

**Table 1:** Indicators urinary tract infections (UTIs) in children in general practice

**Indicator**	**Measurement**	
	**Numerator**	**Denominator**
*First-choice antibiotics*		
1. Antibiotics given	All children receiving antibiotics	All children
2. Amoxicillin/clavulanic acid OR co-trimoxazole	Children receiving first-choice antibiotics	All children receiving antibiotics
*Follow-up*		
3. Episodes with at least one follow-up contact	Number of UTI episodes with >1 contact	All UTI episodes
*Referral*	For all groups: Total referred within the group	Total within the group
4. Children <1 year old		
5. Boys <12 years old		
6. Girls 1–4 years old with second UTI		
7. Girls 5–12 years old with > 1 recurrent UTI		

### Data collection

In the LINH network, the family practice staff routinely records the encoded patient information in electronic medical records (EMR). For the period 2001–2003 data were extracted from the EMR, concerning contacts with the family practice, prescriptions, referrals, and patient characteristics (age, sex).

Practices were excluded from the analyses if they had registered fewer than 46 weeks in 2002 or 2003. Patients who were not on the practice list and patients who had left the practice before 1 January 2004 were also excluded.

### Analyses

The contacts were expressed in units of episodes. Episodes were considered new episodes if the preceding contact for UTI occurred more than 28 days previously. Prescriptions and referrals within 28 days after the last contact for an episode were linked to that episode. The first UTI in 2001 was assumed to be the child's first episode ever. To be able to compare groups, four groups of children were created, based on ICPC code and number of UTI episodes: group 1 (1 episode cystitis), group 2 (2 episodes cystitis), group 3 (≥ 3 episodes cystitis) and group 4 (1 or 2 episodes acute pyelonephritis).

Descriptive statistics were applied to patient characteristics, and the numbers of children receiving medication, follow-up, or referrals. For each indicator, the number of children (or episodes in the case of follow-up) to whom family practitioners (FPs) offered the appropriate care was divided by the total number of children (or episodes in the case of follow-up) needing the provision of such care (Table 1). We calculated the percentage of children with more than one contact during a UTI episode as an indicator of follow-up. Age groups were based on age when having the first UTI.

Student's *t*-test or chi-square tests, as appropriate, were used to investigate whether more boys or girls were treated as recommended by the guideline, and whether younger children were treated more consistently with the guideline than older children. We also investigated whether recurrent childhood UTIs were more often treated according to the guideline than single episodes. We considered a probability level of *P *< 0.05 statistically significant.

## Results

### Study population

**Figure 1:**
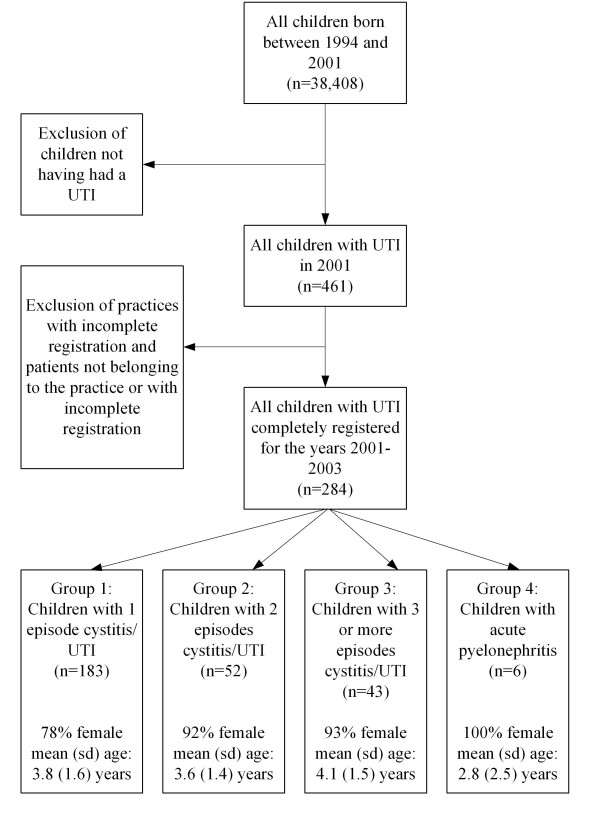
Selection of children with urinary tract infections for the analyses mean (sd) age = age during first episode; sd = standard deviation; UTI = urinary tract infection.

Figure 1 shows the selection of children included in this study. Of 38,408 children in the 120 practices in the year 2001, 461 from 92 practices had a diagnosed UTI (1.2%). Of these 461 children, 284 (62%) in 59 practices could be followed for three years. There were no age or sex differences between included children and children excluded in step 2 of the flow chart.

Of the 284 children included, 278 (98%) were diagnosed with cystitis. Of these children, 66% had one episode (group 1), 19% had two (group 2), and 15% had 3 to 10 (group 3). Six children were diagnosed with acute pyelonephritis (2%; group 4).

During the first UTI episode, the mean age varied from 2.8 (SD 2.5) to 4.1 years (SD 1.5). About 80% of the children in group 1, 90% in groups 2 and 3, and 100% in group 4 were girls (figure 1). Because of the small numbers, no further data are presented for group 4 and for episodes 4–10 in group 3.

### Prescriptions

Table 2 shows that, overall, 66% of the children received antibiotics, varying from 61% to 70%. First choice medication, Amoxicillin/clavulanic acid or co-trimoxazole, was prescribed for 55% to 83% of the children with antibiotics (overall 66%). Another 4% to 25% (overall 13%) of the children with antibiotics received ceftibuten, ofloxacin, or nitrofurantoin. Since childhood UTIs should be treated as complicated UTIs according to the DCGP-guideline, these last three antibiotics should not be prescribed according to the guidelines. Furthermore, 10% to 25% (overall 19%) of the children with antibiotics received amoxicillin without clavulanic acid, which is also not according to the DCGP-guideline. In eight cases, a child who did not receive medication was referred to a medical specialist directly after seeing the GP. No differences in prescription for age or sex were found in any group.

**Table 2:** Prescription of antibiotics in children with urinary tract infections

	**Group 1 ** **(183 children)**	**Group 2** ** (52 children)**	**Group 3** ** (43 children)**	**Total ** **(278 children)**
**Percentage (number) of children/antibiotic prescription**				
Overall children with antibiotics (indicator 1)	66 (121/183)	70 (73/104)	61 (79/129)	66 (273/416)
First episode	66 (121/183)	73 (38/52)	67 (29/43)	68 (188/278)
Second episode	-	67 (35/52)	63 (27/43)	65 (62/95)
Third episode	-	-	53 (23/43)	53 (23/43)
**Percentage (number) of children with antibiotics/choice of antibiotic**				
*Amoxicillin/clavulanic acid or co-trimoxazole*				
Overall first-choice antibiotics (indicator 2)	65 (79/121)	56 (41/73)	76 (60/79)	66 (180/273)
First episode	65 (79/121)	55 (21/38)	69 (20/29)	64 (120/188)
Second episode	-	57 (20/35)	78 (21/27)	66 (41/62)
Third episode	-	-	83 (19/23)	83 (19/23)
*Ceftibuten, ofloxacin, or nitrofurantoin*				
Overall	7 (9/121)	21 (15/73)	14 (11/79)	13 (35/273)
First episode	7 (9/121)	18 (7/38)	14 (4/29)	11 (20/188)
Second episode	-	23 (8/35)	15 (4/27)	19 (12/62)
Third episode	-	-	13 (3/23)	13 (3/23)
*Amoxicillin*				
Overall	25 (30/121)	18 (13/73)	10 (8/79)	19 (51/273)
First episode	25 (30/121)	18 (7/38)	17 (5/29)	22 (42/188)
Second episode	-	17 (6/35)	7 (2/27)	13 (8/62)
Third episode	-	-	4 (1/23)	4 (1/23)

### Follow-up

Twenty-eight percent to 37% of all episodes was followed-up (Table 3). The overall follow-up rate was 32%. About 60% of all episodes with follow-up contacts consisted of two contacts. The second contact took place within 14 days of the first for 90% of the episodes. There were no differences for age or sex groups.

**Table 3:** Follow-up and referrals in children with urinary tract infections

	**Group 1** ** (183 children)**	**Group 2** ** (52 children)**	**Group 3** ** (43 children)**	**Total** ** (278 children)**
**Pecentage (number) of episodes with more than one contact during the episode**				
Overall episodes with more than one contact (indicator 3)	28 (52/183)	34 (35/104)	36 (46/129)	32 (133/416)
First episode	28 (52/183)	35 (18/52)	33 (14/43)	30 (84/278)
Second episode	-	33 (17/52)	37 (16/43)	35 (33/95)
Third episode	-	-	37 (16/43)	37 (16/43)
**Percentage (number) of children with a referral**				
Overall	8 (14/183)	19 (10/52)	33 (14/43)	14 (38/278)
First episode	8 (14/183)	12 (6/52)	9 (4/43)	9 (24/278)
Second episode	-	8 (4/52)	16 (7/43)	12 (11/95)
Third episode	-	-	7 (3/43)	7 (3/43)
**Percentage (number) of children with a referral, special groups of children**				
Children <1 year of age (indicator 4)	67 (2/3)	100 (1/1)	0 (0/0)	75 (3/4)
Boys overall (indicator 5)	18 (7/40)	25 (1/4)	0 (0/3)	17 (8/47)
Girls overall	5 (7/143)	19 (9/48)	38 (15/40)	13 (31/231)
Girls 1–4 years, second episode (indicator 6)	-	9 (2/22)	33 (7/21)	21 (9/43)
Girls 5–9 years, third episode (indicator 7)	-	-	7 (2/27)	7 (2/27)

### Referrals

Table 3 shows that overall 38 of 278 (14%) children were referred for specialist treatment (range: 8–16%). Seventy-six percent was referred to a paediatrician; 8% to a urologist; 3% to radiography; and from 13% it is not clear to what specialist they were referred.

Less than one-third of the children who should have been referred concerning the guideline on referring specific age and/or sex categories, was actually referred. Three of four children younger than one year were referred, and fewer than 25% of the boys were referred during at least one episode. In group 2, 9% of the girls aged 1–4 years were referred during the second episode, and in group 3 33%. Two girls in the 5–9 year olds group (7%) were referred during the third episode. No differences regarding age and sex subgroups were found.

## Discussion

This study showed that the management of childhood UTIs in the Netherlands varied substantially across patients. Only 66% of the children received antibiotics and of these 66% was prescribed first choice antibiotics. There was no follow-up in the majority of the episodes. Referral of children younger than one year was generally consistent with the guidelines, but the referral rates for boys, girls 1–4 years old with a second UTI, and girls 5–12 years old with more than one recurrent UTI should have been much higher, if we consider the guidelines.

We found that the proportion of children receiving amoxicillin decreased proportionally to the number of episodes. Perhaps FPs prescribe amoxicillin routinely because this medication is much older than the combined form with clavulanic acid, and has less side effects than amoxicillin/clavulanic acid. If amoxicillin alone does not work, they prescribe the combination. Prescribing ceftibuten, ofloxacin, or nitrofurantoin suggests that not all FPs are aware that, according to the guidelines, childhood UTI should be treated as complicated UTI. This is confirmed by the fact that, for 98% of all children, the FP had registered the ICPC for cystitis instead of the code for complicated UTI (pyelonephritis). No significant differences of age or gender might imply that GPs are unaware of the increased risk of complications or underlying pathology in boys and younger children. Such unawareness may lead to health complications when the child is older.

The LINH network provided a unique opportunity for collecting prospective data regarding clinical management in routine healthcare settings, but one can question FP registration behaviour and whether all childhood UTIs were identified with the ICPC codes 'acute pyelonephritis' and 'cystitis'. However, the incidence we found for 0 to 6-year-olds, which is 12.0 (461*1000/38408), is comparable to those in other Dutch studies: 15.1 [10] and 13.2 [14]. Since direct observation and hand-searching medical records are infeasible, using databases of consultation registrations seems to be the optimal method for collecting information about FP clinical behaviour.

It is difficult to compare our results on FP management with other studies because our data are prospectively collected, had a follow-up period of three years, and focussed on primary care and individual young children; this in contrast to other studies. A study by Kwok et al. [15] already gave some insight in the management of children's UTIs in Dutch family practice. But compared to our study, this study concentrated on a much wider age range, although the children most vulnerable to renal scarring are the younger ones. The study also had a follow-up period of only one year and did not pay attention to follow-up after the antiobiotic treatment. One British study found that 37% of children with proven UTI were sent for renal tract imaging [16], and another Dutch study reported 4% of the children being referred [14]. Two other British studies found much higher rates of referral [17,18]. However, these last two studies used postal questionnaires to measure FP behaviour, whereas our study and the first two studies used medical records. Reporting behaviour retrospectively may lead to overestimation of guideline adherence because of social desirability bias [19].

Improvement of professional performance might substantially improve clinical outcomes. This is demonstrated in Sweden, where a more aggressive approach led to no new cases of uraemia caused by non-obstructive pyelonephritis during the years 1986–1995 [20]. But, development and distribution of guidelines do not necessarily lead to better patient care [21]. Future research could focus on developing interventions to improve prescription, follow-up, and referrals, but should also consider motives for not following the guidelines. Because not many childhood UTIs appear in family practice in the Netherlands – our study saw an average of five children per practice in one year – interventions should not be too time consuming for the FPs.

## Conclusion

In order to prevent negative health outcomes, treatment of childhood urinary tract infections in Dutch family practice should be improved with respect to prescription, follow-up, and referral. Quality improvement should address the low incidence of urinary tract infections in children in family practice.

## Abbreviations

DCGP = Dutch College of General Practitioners; EMR = electronic medical record; FP = family practitioner; ICPC = International Classification of Primary Care; LINH = Netherlands Information Network of General Practice; UTI = urinary tract infection

## Competing interests

The author(s) declare that they have no competing interests.

## Authors' contributions

MH had primary responsibility for protocol development, analyses, and writing the manuscript. MW, JCCB, RJW, and JCvdW participated in the development of the protocol and contributed to the writing of the manuscript. RG supervised the design and execution of the study and contributed to the writing of the manuscript. All authors have read and approved the final manuscript.

## Pre-publication history

The pre-publication history for this paper can be accessed here:


